# The effect of aspirin on kidney allograft outcomes; a short review to current studies

**DOI:** 10.15171/jnp.2017.19

**Published:** 2017-01-30

**Authors:** Wisit Cheungpasitporn, Charat Thongprayoon, Donald G. Mitema, Michael A. Mao, Ankit Sakhuja, Wonngarm Kittanamongkolchai, Maria L Gonzalez-Suarez, Stephen B. Erickson

**Affiliations:** ^1^Division of Nephrology and Hypertension, Mayo Clinic, Rochester, Minnesota, USA; ^2^Division of Pulmonary and Critical Care Medicine, Mayo Clinic, Rochester, Minnesota, USA

**Keywords:** Aspirin, Kidney transplantation, Delayed graft function, Allograft failure, Transplantation

## Abstract

**Context:**

The use of aspirin in chronic kidney disease (CKD) patients has been shown to reduce myocardial infarction but may increase major bleeding. However, its effects in kidney transplant recipients are unclear.

**Evidence Acquisitions:**

A literature search was performed using MEDLINE, EMBASE, and Cochrane Database of Systematic Reviews from inception through September 2016. We included studies that reported odd ratios, relative risks or hazard ratios comparing outcomes of aspirin use in kidney transplant recipients. Pooled risk ratios (RR) and 95% confidence interval (CI) were assessed using a random-effect, generic inverse variance method.

**Results:**

We included 9 studies; enrolling 19759 kidney transplant recipients that compared aspirin with no treatment. Compared to no treatment, aspirin reduced the risk of allograft failure (4 studies; RR: 0.57, 95% CI: 0.33 to 0.99), allograft thrombosis (2 studies; RR: 0.11, 95% CI: 0.02 to 0.53), and major adverse cardiac events (MACEs) or mortality (2 studies; RR: 0.72, 95% CI: 0.59 to 0.88), but not allograft rejection (3 studies; RR: 0.86, 95% CI: 0.45 to 1.65) or delayed graft function (DGF) (2 studies; RR: 1.00, 95% CI: 0.58 to 1.72) in kidney transplant recipients. The data on risk of major or minor bleeding were limited.

**Conclusions:**

Our meta-analysis demonstrates that administration of aspirin in kidney transplant recipients is associated with reduced risks of allograft failure, allograft thrombosis, and MACEs or mortality, but not allograft rejection or DGF. Future studies are needed to assess the risk of bleeding, and ultimately weigh the overall risks and benefits of aspirin use in specific kidney transplant patient populations.

**Implication for health policy/practice/research/medical education:**

The effects of aspirin use on allograft outcomes are unclear. In this meta-analysis including 9 studies with 19 759 kidney transplant recipients, we demonstrate significant associations between the use of aspirin and a 0.57-fold reduced risk of allograft failure, 0.11-fold reduced risk of allograft thrombosis and 0.72-fold reduced risk of major adverse cardiac events (MACEs) or mortality.

## 1. Background

The administration of aspirin in patients with chronic kidney disease (CKD) has been shown to provide similar potential benefit for cardiovascular risk reduction in CKD and non-CKD patients (1). Despite the fact that the use of aspirin and other antiplatelet agents in CKD patients may reduce myocardial infarction, data from randomized controlled trials (RCTs) showed that antiplatelet agents also significantly increased major bleeding in CKD patients (1-3). Thus, the use of aspirin in CKD patients may outweigh harms among patients with low annual risks of cardiovascular events (2).

In kidney transplant recipients, notwithstanding significant improvements in short-term kidney allograft survival (4), long-term graft and patients survivals are still an ongoing concern (5,6). In addition, cardiovascular disease remains the most common cause of death after kidney transplantation worldwide (7,8). Previous studies of kidney transplant recipients have shown no significant benefits of antiplatelet agents, including dipyridamole and picotamide, in the reduction of allograft rejection or improvement of allograft survival (2,9-11).

The effects of aspirin administration on allograft outcomes including allograft thrombosis, delayed graft function (DGF), acute/chronic allograft rejection and major adverse cardiac events (MACEs) or mortality, however, are not clearly demarcated. A few studies have demonstrated these beneficial effects of aspirin in kidney transplant recipients (12-15). Conversely, several studies have shown no significant effects (16-20) in kidney transplant population. Thus, we conducted a meta-analysis to assess the effects of aspirin on these kidney allograft outcomes.

## 2. Evidence Acquisition

### 2.1. Search strategy

W.C. and C.T. (two investigators) independently searched published articles and conference abstracts listed in EMBASE, MEDLINE and the Cochrane database from inception through September 2016 using the following words: “aspirin” AND “transplantation” AND “kidney” or “renal” (Item S1 in online supplementary data). A manual search for additional relevant studies using references from retrieved articles was also performed. Differing decisions were resolved by mutual consensus.

### 2.2. Inclusion criteria and outcomes

The inclusion criteria were 1) RCTs or observational studies published as original studies or conference abstracts that assessed the effects of aspirin in kidney transplant populations, 2) studies that presented data to calculate relative risks, hazard ratios, or standardized incidence ratios with 95% confidence intervals (CI), and 3) a reference group composed of patients who were not on treatment with aspirin as control group.

Our outcomes of interest in this study included allograft failure, allograft thrombosis, allograft rejection, DGF and MACEs or mortality. The quality of each study was evaluated by using the Jadad quality-assessment scale (21) for RCTs and the Newcastle-Ottawa quality assessment scale (22) for observational studies.

### 2.3. Data extraction

A standardized data collection form was utilized to extract the following information: study design, last name of first author, title of article, year of study, country of origin, year of publication, sample size, definition of aspirin use and control groups, and point of outcome assessment.

### 2.4. Statistical analysis

Review manager software (version 5.3) from the Cochrane collaboration was utilized for data analysis. Point estimates and standard errors were obtained from individual studies and were consolidated by the generic inverse variance method of DerSimonian and Laird (23). A random-effect model was employed rather than a fixed-effect model, given the high likelihood of between-study variances. Statistical heterogeneity was appraised using Cochran’s Q test. This statistic was complemented with the I^2^ statistic, which quantifies the proportion of the total variation across studies that is due to heterogeneity rather than chance. An I^2^ of 0%–25% renders insignificant heterogeneity, 26%–50% low heterogeneity, 51%–75% moderate heterogeneity and >75% high heterogeneity (24). The possibility of publication bias was evaluated by funnel plots of the logarithm of odds ratios vs. their standard errors (25).

## 3. Results

The search strategy yielded 2225 potentially relevant articles: 2117 were excluded based on the title and abstract which apparently showed that they did not fulfill inclusion criteria regarding study design, article type, population, or outcome of interest (Item S2). The remaining 108 articles underwent full-length review, with 99 excluded because they were not observational studies or RCTs (n = 12) or did not report outcomes of interest (n = 87). Nine cohort studies with 19 759 kidney transplant recipients that compared aspirin with no treatment were included in the meta-analysis. Table 1 contains detailed characteristics and quality assessment of all included studies.

**Table 1:** Main characteristics of the observational studies included in this meta-analysis

	Abendroth et al (16)	Taha et al (17)	Robertson et al (12)
Country	Germany	UK	UK
Study design	Cohort study	Cohort study	Cohort study
Year	1997	2000	2000
Total number‏	176	226	955
Study sample	Kidney transplant patients	Cadaveric kidney transplant patients	Cadaveric and living related kidney transplant patients receiving cyclosporine-based tripple immunosuppression
Exposure definition	0.5 g aspirin prior to declamping	Daily aspirin 150 mg for the first 3 postoperative months	Aspirin 75-150 mg once daily for 1 month post-transplant or long-term for high-risk patients
Adjusted OR or RR for outcome	Rejection 1.65 (0.91-3.00) 36-month graft failure 0.71 (0.31-1.61) 36-month mortality 0.48 (0.14-1.64)	Primary allograft thrombosis 0 2-year graft failure 0.99 (0.50-1.96) Chronic rejection 0.54 (0.28-1.04)	Renal vein thrombosis 0.21 (0.09-0.51)
Confounder adjusted	Matched for age, gender, cold ischemia time, HLA-match, immunosuppressive regimen, and panel-reactive antibodies	None	None
Quality assessment (Newcastle-Ottawa scale)	Selection:3 Comparability: 2 Outcome: 2	Selection:3 Comparability: 0 Outcome: 2	Selection:2 Comparability: 0 Outcome: 2
	Murphy et al (18)	Grotz et al (13)	Oien et al (19)
Country	UK	Germany	Northern Europe and Canada
Study design	Cohort study	Cohort study	Cohort study
Year	2001	2004	2006
Total number	226	830	1052
Study sample	Cadaveric kidney transplant patients	Kidney transplant patients	Kidney transplant patients
Exposure definition	Aspirin 150 mg daily for the first 3 months after transplant	Aspirin 100 mg daily	Aspirin use at baseline
Adjusted OR or RR for outcome	Delayed graft function 1.18 (0.67-2.07) Acute rejection 0.71 (0.41-1.24)	Graft failure; defined as death of the patient or the need to return to chronic dialysis therapy 0.44 (0.32-0.61)	Non-fatal MI or cardiac death 1.36 (0.81-2.28)
Confounder adjusted	None	Statin, number of antihypertensive medications, gender, donor type, acute allograft rejection, age, number of transplantation, period of transplantation, HLA mismatch, warm ischemia time, cold ischemia time and renal diseases	None
Quality assessment (Newcastle-Ottawa scale)	Selection:2 Comparability: 0 Outcome: 2	Selection:4 Comparability: 2 Outcome: 3	Selection:4 Comparability: 0 Outcome: 3
	Stechman et al (14)	Pilmore (15)	Esfandiar et al (20)
Country	UK	Multi-center	Iran
Study design	Cohort study	Cohort study	Cohort study
Year	2007	2011	2012
Total number	797	15 410	87
Study sample	Cadaveric and living related kidney transplant patients	Kidney transplant patients	Pediatric kidney transplant patients
Exposure definition	Aspirin 75 mg daily for 28 days following transplant	Anti-platelet use within 4 months and 12 months post-transplant	Heparin 50 units/kg every 8 hours for 7 days and aspirin 5 mg/kg three times a week from day 3 of transplant for 3 months
Adjusted OR or RR for outcome	Renal vein thrombosis 0.04 (0.01-0.30)	Major adverse cardiac events At 12 months 0.95 (0.67-1.36) At 5 years 0.73 (0.60-0.89)	Thrombosis 0 Graft failure 0.14 (0.02-1.12) Acute tubular necrosis 1.44 (0.50-4.17)
Confounder adjusted	None	Age, race, BMI, cause of renal failure, time on dialysis, panel reactive antibodies, history of cardiovascular disease risk factors	Matched for age and sex
Quality assessment (Newcastle-Ottawa scale)	Selection:2 Comparability: 0 Outcome: 2	Selection:4 Comparability: 2 Outcome: 3	Selection:3 Comparability: 1 Outcome: 2

### 3.1. Effects of aspirin on kidney allograft outcomes

**Figure 1:**
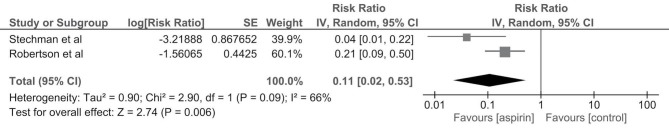
Forest plot of all included studies comparing risk of allograft thrombosis in kidney transplant recipients who received aspirin vs control; square data markers, RRs; horizontal lines, 95% CIs, with marker size reflecting statistical weight of study using random-effects meta-analysis. Diamond data markers, overall RRs and 95% CIs for outcomes of interest. IV, inverse variance; SE, standard error.

**Figure 2:**
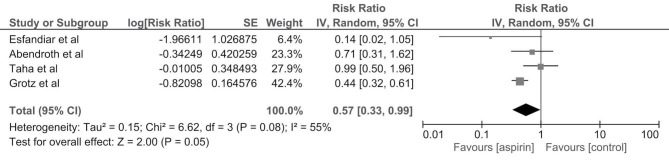
Forest plot of all included studies comparing risk of allograft failure in kidney transplant recipients who received aspirin vs control; square data markers, RRs; horizontal lines, 95% CIs, with marker size reflecting statistical weight of study using random-effects meta-analysis. Diamond data markers, overall RRs and 95% CIs for outcomes of interest. IV, inverse variance; SE, standard error

The pooled risk ratio (RR) of allograft thrombosis in recipients (2 studies) who received aspirin was 0.11 (95% CI: 0.02-0.53, *I*^2^ = 66%) as shown in Figure 1. However, compared to no treatment, aspirin did not significantly reduce the risk of DGF with pooled RR (2 studies) of 1.00 (95% CI: 0.58-1.72, *I*^2^ = 0%) (Figure S1) or acute/chronic allograft rejection with pooled RR (3 studies) of 0.86 (RR: 0.86, 95% CI: 0.45-1.65,* I*^2^ = 71%) (Figure S2). Nevertheless, aspirin significantly decreased the risk of allograft failure (4 studies; RR: 0.57, 95% CI: 0.33 to 0.99, *I*^2^ = 55%) as shown in Figure 2.

### 3.2. Effects of aspirin on major adverse cardiac events or mortality

The pooled RR of MACEs or mortality in recipients (3 studies) who received aspirin was 0.86 (95% CI: 0.52-1.43, *I*^2^ = 63%) (Figure S3). When meta-analysis was limited only to the studies with adjusted analysis to minimize the effects of confounders, the pooled RR of MACEs or mortality was 0.72 (95% CI: 0.59-0.88, *I*^2^ = 0%) (Figure S4).

### 3.3. Evaluation for publication bias

Funnel plots to evaluate publication bias regarding the risks of allograft failure and MACEs or mortality in recipients using aspirin are shown in Figure S5-6. Overall, the publication bias was insignificant. However, due to the limited number of studies, the power of the test was too low to distinguish chance from real asymmetry (26).

## 4. Discussion

In this meta-analysis of 19 759 kidney transplant patients, we demonstrated significant associations between the use of aspirin and a 0.57-fold reduced risk of allograft failure, 0.11-fold reduced risk of allograft thrombosis and 0.72-fold reduced risk of MACEs or mortality. However, the use of aspirin did not significantly decrease DGF or allograft rejection. In addition, the data on risk of major or minor bleeding in recipients with aspirin use were limited.

Atherosclerosis is an important and common component in the pathogenesis of chronic allograft failure (27,28). Chronic transplant vasculopathy and atherosclerosis are both recognized as special forms of inflammatory reactions within the vessel wall (27-29). Platelets have long been identified as major determinants, especially in the process of plaque rupture and vessel occlusion or stenosis (13,29). In addition, the degree of transplant vasculopathy is also correlated with the activation status of platelets (30,31). Studies have identified soluble CD40L as an important link between platelet activation and inflammation (32-34), and the blockade of the CD40 and CD40L system has been shown to suppress allograft transplant arteriopathy (31). Additionally, Grotz et al (13) demonstrated that the positive impact of aspirin treatment on long-term kidney transplantation outcome was associated with the duration of aspirin treatment (13). Therefore, our meta-analysis confirmed associations between the use of aspirin and reduced risks of allograft thrombosis and allograft failure in kidney transplant recipients.

Aspirin has been proved to be effective in primary and secondary prevention of cardiovascular disease complications (35,36). Since kidney transplant recipients are considered as moderate to high risk for cardiovascular diseases (37), not surprisingly, our studies also showed an association between reduced risk of MACEs or mortality. Despite the benefits of aspirin, studies have demonstrated that antiplatelet agents could be harmful with significantly increased risk of major bleeding in CKD patients (1-3). Unfortunately, the data on risks of major or minor bleeding associated with aspirin use in kidney transplant recipients were unclear. Although a study of 15 181 percutaneous core biopsies (including a kidney biopsies) showed that the incidence of bleeding in patients receiving aspirin within 10 days before biopsy and those not taking aspirin was not significantly different (38), the data on other types of bleeding such as gastrointestinal (GI) bleeding are limited. In the general population, a recent meta-analysis demonstrated that GI bleeding with low-dose aspirin had an incidence of 0.48-3.64 cases per 1000 person-years and an overall 1.4-fold increased risk (39). In addition, compared with aspirin alone, an increased bleeding risk was observed with use of aspirin combined with non-steroidal anti-inflammatory drugs, clopidogrel and selective serotonin reuptake inhibitors (39).

There are several limitations of our meta-analysis. First, this current study is a meta-analysis of observational studies. Thus, a causal relationship needs to be cautiously interpreted. Second, the majority of the included studies did not have available kidney allograft biopsy information, and consequently, the cause of allograft dysfunction or allograft failure was not known. However, from all available data, it is most likely that the association between aspirin use and reduced risk of allograft failure is through lowered risk of transplant vasculopathy and atherosclerosis (13,31-34). Finally, studies have shown reduced patient survival in kidney transplant recipients with elevated cardiac troponin T (cTnT) (40,41). The data on the risk and benefits of aspirin especially in these high-risk transplant populations, however, were lacking in the included studies in our meta-analysis.

## 5. Conclusions

In summary, this meta-analysis shows reduced risks of allograft failure, allograft thrombosis and MACEs or mortality, but not allograft rejection or DGF among renal transplant recipients treated with aspirin. Ultimately to weigh the overall risks and benefits of aspirin use in specific kidney transplant patient populations, future studies assessing the major or minor bleeding risks in aspirin treated kidney transplant recipients are required.

## Conflicts of interest

The authors declare that they have no conflicting interest.

## Authors’ contributions

All authors had access to the data and a role in writing the manuscript. All authors read and signed the final paper.

## Funding/Support

None.

## Supplementary Materials

Supplementary Materials Supplementary Data contains Item S1, Item S2, and Figures S1-S6.
