# Supplementary material for: The effect of aspirin on kidney allograft outcomes; a short review to current studies
Source: J Nephropathol. 2017 Jan 30;6(3):110–7. doi: 10.15171/jnp.2017.19 (PMC5607969; doi:10.15171/jnp.2017.19)

Online Supplementary Data

Item S1: Search Strategy

Database: Ovid, MEDLINE, Cochrane Database of Systematic Reviews, Cochrane Central Register of Controlled Trials

1. exp aspirin/
2. aspirin$.mp.
3. 1 or 2
4. exp transplantation/
5. transplantation$.mp.
6. 4 or 5
7. kidney$.mp.
8. renal$.mp.
9. 7 or 8
10. 6 and 9
11. 3 and 10
12. limit 24 to all adult
13. limit 25 to humans

Item S2: Outline of our search methodology.

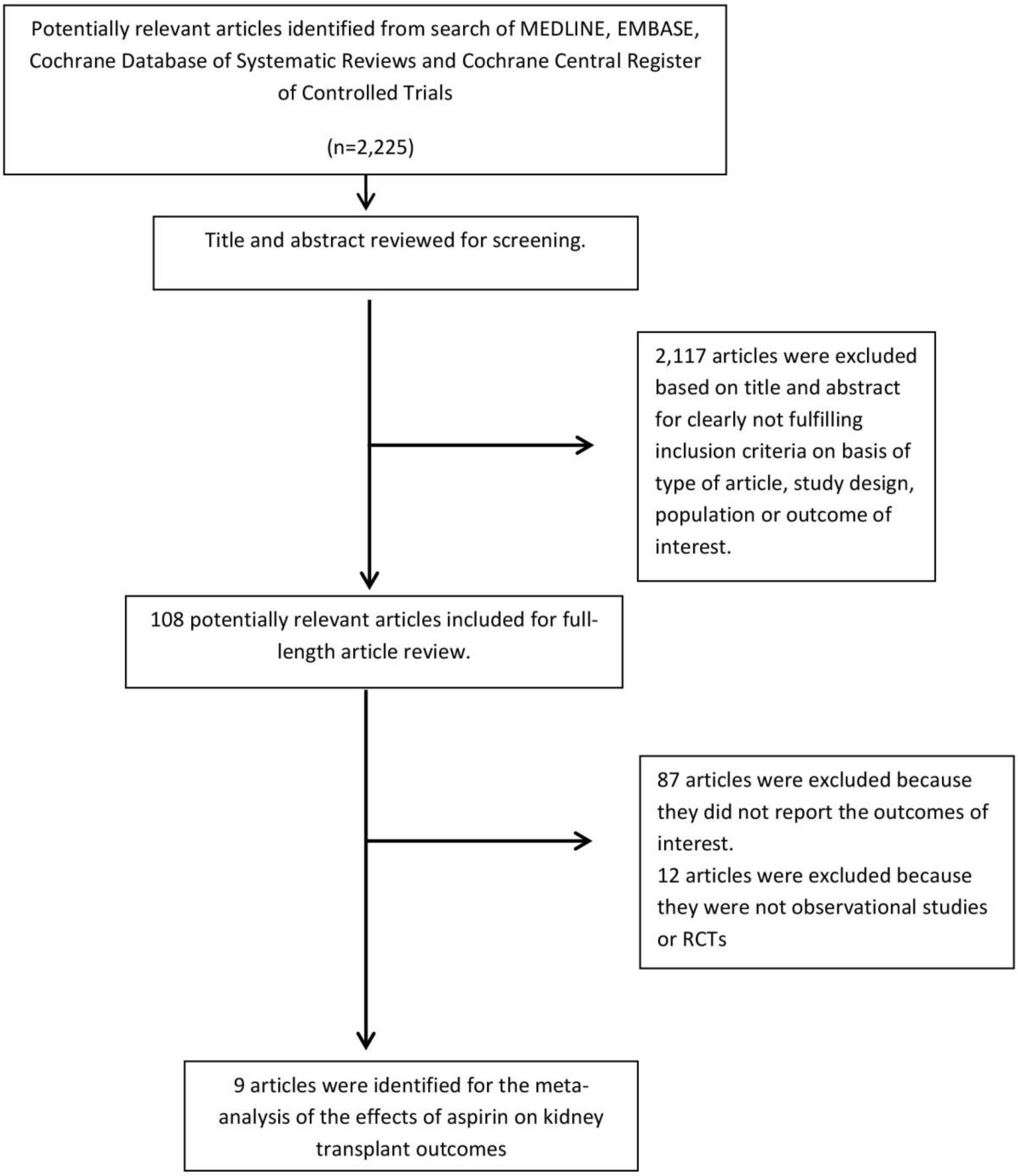

Figure S1: Forest plot of all included studies comparing DGF risk in recipients who received aspirin and those who did not; square data markers represent risk ratios (RRs); horizontal lines, the 95% CIs with marker size reflecting the statistical weight of the study using random-effects meta-analysis. A diamond data marker represents the overall RR and 95% CI for the outcome of interest.

Study or Subgroup	log[Risk Ratio]	SE	Weight	Risk Ratio IV, Random, 95% CI
Esfandiar et al	0.364643	0.541088	22.0%	1.44 [0.50, 4.16]
Murphy et al	0.165514	0.287762	78.0%	1.18 [0.67, 2.07]
**Total (95% CI)**			**100.0%**	**1.23 [0.75, 2.03]**

Heterogeneity: $Tau^2 = 0.00$; $Chi^2 = 0.11$, df = 1 (P = 0.75); $I^2 = 0\%$
Test for overall effect: Z = 0.82 (P = 0.41)

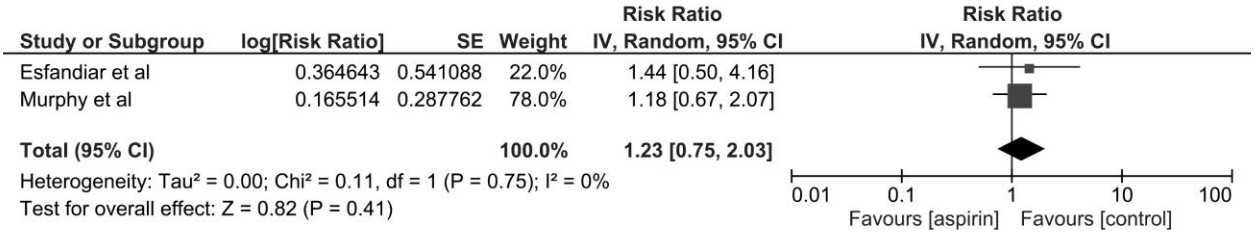

Figure S2: Forest plot of all included studies comparing risk of acute/chronic allograft rejection in recipients who received aspirin and those who did not; square data markers represent risk ratios (RRs); horizontal lines, the 95% CIs with marker size reflecting the statistical weight of the study using random-effects meta-analysis. A diamond data marker represents the overall RR and 95% CI for the outcome of interest.

Study or Subgroup	log[Risk Ratio]	SE	Weight	Risk Ratio IV, Random, 95% CI
Abendroth et al	0.500775	0.304317	33.5%	1.65 [0.91, 3.00]
Murphy et al	-0.34249	0.282324	34.9%	0.71 [0.41, 1.23]
Taha et al	-0.61619	0.334741	31.6%	0.54 [0.28, 1.04]
**Total (95% CI)**			**100.0%**	**0.86 [0.45, 1.65]**

Heterogeneity: $Tau^2 = 0.23$; $Chi^2 = 6.97$, df = 2 (P = 0.03); $I^2 = 71\%$
Test for overall effect: Z = 0.44 (P = 0.66)

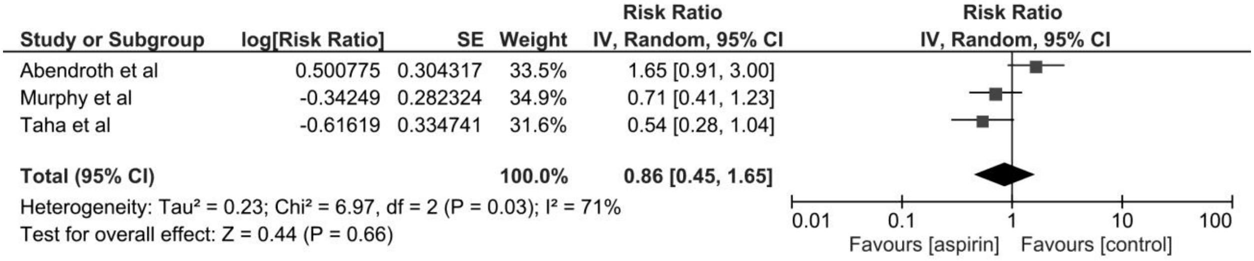

Figure S3: Forest plot of all included studies comparing risk of MACE or mortality in recipients who received aspirin and those who did not; square data markers represent risk ratios (RRs); horizontal lines, the 95% CIs with marker size reflecting the statistical weight of the study using random-effects meta-analysis. A diamond data marker represents the overall RR and 95% CI for the outcome of interest.

Study or Subgroup	log[Risk Ratio]	SE	Weight	Risk Ratio IV, Random, 95% CI
Abendroth et al	-0.73397	0.627757	12.9%	0.48 [0.14, 1.64]
Oien et al	0.307485	0.264004	35.3%	1.36 [0.81, 2.28]
Pilmore et al	-0.31471074	0.100585	51.8%	0.73 [0.60, 0.89]
**Total (95% CI)**			**100.0%**	**0.86 [0.52, 1.43]**

Heterogeneity: Tau² = 0.12; Chi² = 5.47, df = 2 (P = 0.07); I² = 63%
Test for overall effect: Z = 0.58 (P = 0.56)

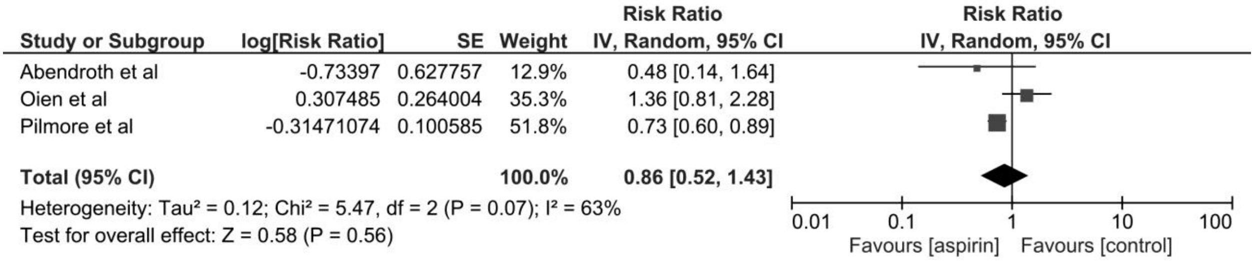

Figure S4: Forest plot of all included studies limited only to the studies with adjusted analysis comparing risk of MACE or mortality in recipients who received aspirin and those who did not; square data markers represent risk ratios (RRs); horizontal lines, the 95% CIs with marker size reflecting the statistical weight of the study using random-effects meta-analysis. A diamond data marker represents the overall RR and 95% CI for the outcome of interest.

Study or Subgroup	log[Risk Ratio]	SE	Weight	Risk Ratio IV, Random, 95% CI
Abendroth et al	-0.73397	0.627757	2.5%	0.48 [0.14, 1.64]
Oien et al	0.307485	0.264004	0.0%	1.36 [0.81, 2.28]
Pilmore et al	-0.31471074	0.100585	97.5%	0.73 [0.60, 0.89]
**Total (95% CI)**			**100.0%**	**0.72 [0.59, 0.88]**

Heterogeneity: Tau² = 0.00; Chi² = 0.43, df = 1 (P = 0.51); I² = 0%
Test for overall effect: Z = 3.27 (P = 0.001)

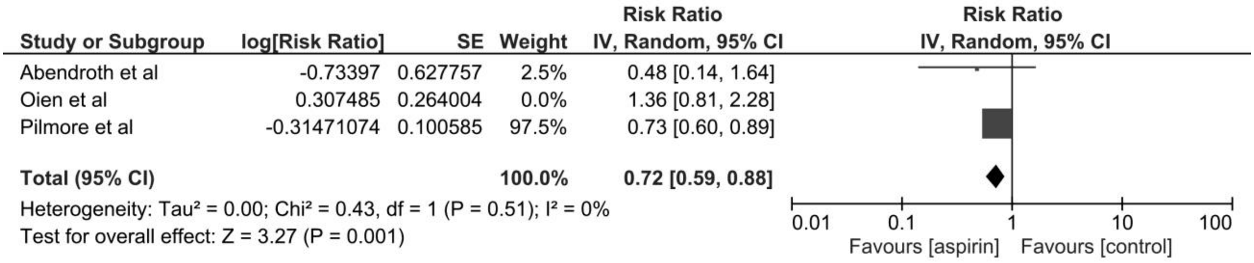

Figure S5: Funnel plot of included studies in the meta-analysis for risk of renal allograft failure in patients who received aspirin. RR = risk ratio, SE = standard error.

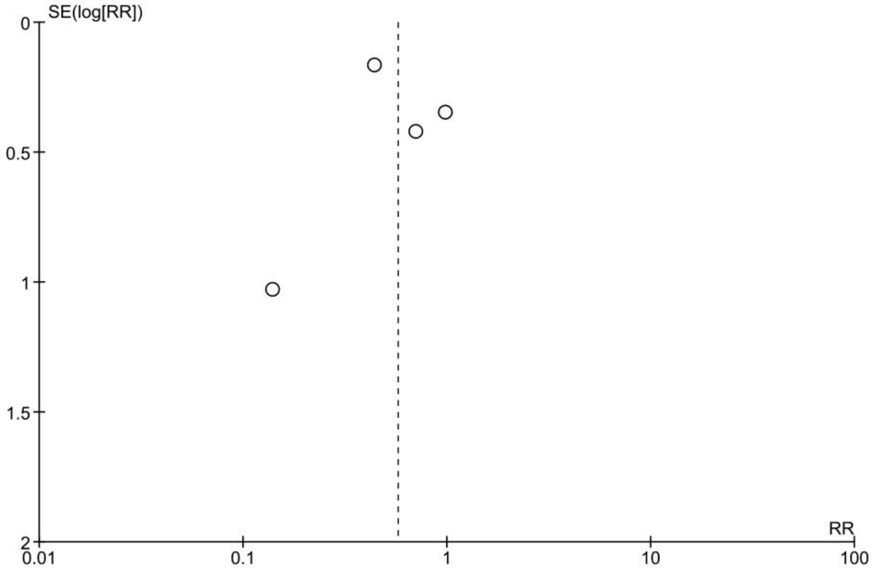

Figure S6: Funnel plot of included studies in the meta-analysis for risk of MACE or mortality in patients who received aspirin. RR = risk ratio, SE = standard error.

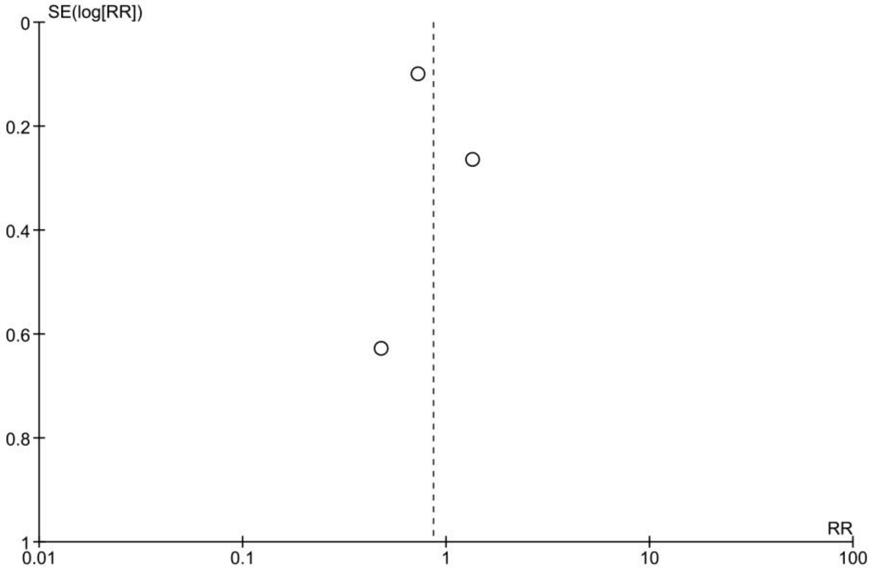

Supplement: Supplementary Materials — Supplementary Data contains Item S1, Item S2, and Figures S1-S6. [file jnp-6-110-s001.pdf]
